# Flavor education and training in olfactory dysfunction: a pilot study

**DOI:** 10.1007/s00405-020-05950-8

**Published:** 2020-04-04

**Authors:** Gerold Besser, Michaela M. Oswald, David T. Liu, Bertold Renner, Christian A. Mueller

**Affiliations:** 1 Department of Otorhinolaryngology and Head and Neck Surgery, Medical University of Vienna, Währinger Gürtel 18-20, 1090 Vienna, Austria; 2 Institute of Experimental and Clinical Pharmacology and Toxicology, Friedrich-Alexander Universität Erlangen-Nürnberg, Erlangen, Germany; 3 Institute of Clinical Pharmacology, Medical Faculty Carl Gustav Carus, Technische Universität Dresden, Dresden, Germany

**Keywords:** Anosmia, Hyposmia, Olfactory training, Quality of life, Retronasal

## Abstract

**Purpose:**

Olfactory training is recommended in olfactory dysfunction (OD) showing promising results. OD patients frequently ask for training modifications in the hope of a better outcome. Also, a lack of knowledge of the flavor system is evident. This investigation sought to implement flavor education (FE) and encourage patients to experience flavors in terms of a flavor training (FT).

**Methods:**

In included patients (*n* = 30), OD was either of postinfectious (86.7%) or posttraumatic (13.3%) cause. Chemosensory abilities were tested orthonasally (using Sniffin Sticks = TDI) and retronasally (using the Candy Smell Test = CST). Key points of flavor perception were demonstrated in an educative session. Subjects were instructed to consciously experience flavors out of a list of 50. Effects of FT were explored in two groups (group A and B), with group B starting FT 17 weeks later.

**Results:**

FE was appreciated and drop-out rate stayed very low (one participant). Compliance was high and 30.4 ± 12.9 flavors were tried. Overall TDI scores improved in 10 patients (6 group A, 4 group B) in a clinically significant way (> 5.5). For group A (starting FT earlier) rm-ANOVA showed a significant effect of session (timepoint) on CST (*p* < 0.01).

**Conclusion:**

Flavor education is demonstrated as feasible and appreciated in a clinical setting. FT seems to be a welcomed second-line therapy in patients with olfactory dysfunction. This study shows beneficial trends of FT; however, further studies with larger sample sizes and standardized training protocols are needed.

## Introduction

Decreased chemosensory abilities (in particular olfactory abilities) are still an underestimated burden. Food enjoyment strongly relies on olfactory sensations [1] and dietary changes have been reported in olfactory dysfunction (OD) using a questionnaire-based tool [2]. Frequently, OD patients are not aware of the important contribution of the sense of smell to flavor perception [3]. They report “taste dysfunction”, although olfactory function (i.e., flavor perception) is reduced [4, 5]. This lack of knowledge of the flavor system often complicates a clinical work-up in OD patients.

Another challenge in smell and taste clinics and beyond: the degree of suffering in OD is variable and often unpredictable. Congenital anosmia patients frequently report few to no disease related complaints [6, 7]. OD patients with a postinfectious or posttraumatic cause (with a sudden onset), however, often are tremendously desperate about missing perceptive capacity. Also, the degree of complaint can be more pronounced in postinfectious patients compared to OD patients of sinonasal cause [8]. In contrast to the cause of OD, retronasal olfactory function may be significantly predictive for quality of life [9]. Educative lessons on the flavor system and routine retronasal olfactory testing (as various tools have been published [10–15]) therefore seem to be valuable and should be implemented whenever possible.

Furthermore, in OD patients an urge for novel therapy options is evident with promising research progress on alternative therapy strategies [7, 16, 17]. Nevertheless, to date olfactory training (OT) remains the single evidence-based treatment option in non-sinunasal (i.e., sensorineural) OD [18–21]. Adding budesonide irrigation, changing odors and prolonging training may increase its beneficial potentials [22, 23], whereas more complex odors do not seem to influence training outcome [24]. OD patients frequently very precisely ask for possible modifications of OT in the hope of positive effects. In the context of the flavor system, we hypothesize retronasal olfactory training (i.e., flavor training, FT) to be a potential beneficial modification or “add-on” therapy to orthonasal OT.

The aim of the following pilot study hence was to implement sensory education (i.e., flavor education, FE) in our work-up and evaluate feasibility and acceptance of FT in OD patients.

## Materials and methods

### Subjects

Patients with subjective OD were prospectively recruited through our smell and taste clinic. Data collection was performed from January 2012 to November 2015. Rhinologic examination, the patients’ history and, if necessary imaging were used to determine possible reasons.

Thirty patients, 21 females and 9 males, with a mean age (mean ± standard deviation/SD) of 58.1 ± 12.0 years (range 28–74 years) were included in this study. Predominant reason was postinfectious (26 patients, 86.7%), whilst four patients suffered from posttraumatic OD (13.3%). According to summed scores of odor threshold (*T*), discrimination (*D*), and identification (*I*) 6 (20%) patients were anosmic, 21 (70%) were hyposmic and 3 (10%) participants were normosmic at first visit. The mean duration of OD was 13.2 ± 7.5 months (range 3–24 months). Patients’ characteristics per group (see below) are shown in Table 1.

**Table 1:** Patients’ characteristics and results at first visit

	A (*n* = 15)		B (*n* = 15)		All (*n* = 30)	
Gender	f 11	m 4	f 10	m 5	f 21	m 9
ReasOD	I 13	T 2	I 13	T 2	I 26	T 4
	Mean	SD	Mean	SD	Mean	SD
Age	58.9	13.6	59.3	10.5	58.1	12.0
DurOD	14.7	8.0	11.6	7.0	13.2	7.5
*T*	4.6	2.7	4.3	2.8	4.5	2.7
*D*	8.9	2.9	9.1	2.0	9.0	2.4
*I*	9.8	3.3	8.6	3.3	9.2	3.3
TDI	23.3	6.7	22.1^a^	6.2	22.7^c^	6.4
CST	14.3	2.3	14.3^a,b^	2.9	14.3^c^	2.6
SAS	3.1	1.9	2.8	1.0	2.9	1.5
SAF	6.3	2.6	5.5^b^	2.3	5.9	2.4
DAS	6.0	6.0	7.0	7.0	6.0	6.5
SF-36	101	4.7	103	3.9	102	4.3

*CST* candy smell test, *DAS* Dietary alterations score, *DurOD* duration of olfactory dysfunction in months, *f* female, *m* male, *I* postinfectious, *ReasOD* reason for olfactory dysfunction, *SAS/F* subjective assessment of smell and flavor, *SF-36* short form health related questionnaire, *T* posttraumatic, *TDI* odor threshold, discrimination and identification score

^a–c^Symbols indicate significantly correlating pairs (all *p* < 0.05, see results section in text)

### Timeline

**Fig. 1:**
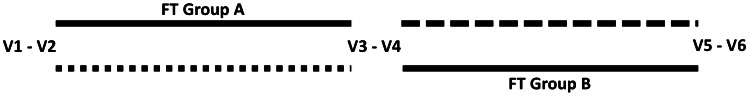
Illustration of applied delayed-start study design. After the 2nd visit (V2) group A performed flavor training (FT) for 16 weeks and were allowed to continue after the 4th visit (dashed line). Dotted line: Group B performed no structured training before V4. V1–V6: 1st to 6th visit (overall 35 weeks)

For each subject study duration (V1-6) was 35 weeks (see Fig. 1 for visualization of timeline). To evaluate feasibility and acceptance of FT all enrolled subjects were assigned for FT in this pilot study. Starting points of FT, however, were altered to partly compensate for a control group and evaluate trends in effectiveness of FT (by attempting a delayed-start study design [25]). Therefore, subjects were randomized into two groups (A and B). FE was conducted at V2 (after 1 week) for group A and at V4 (after 18 weeks) for group B and FT started right after FE. FT lasted for 16 weeks. Olfactory testing was performed at V1, V3 and V5. In-between V1 and V2, V3 and V4, as well as V5 and V6 were 1 week. In case of V2 for group B and V4 for group A, there was time for questions and questionnaire collection. V6 served as a closure consultation.

### Olfactory tests

For *orthonasal testing* we examined TDI (sum of T, D and I) using Sniffin’ Sticks (Burghart GmbH, Wedel, Germany). These felt-tip pens with odorants are widely used and large population normative datasets are available [26]. Administration of the three subtests is described in detail elsewhere [27–29]. The TDI score can be used to categorize anosmia (16 or less), hyposmia (more than 16, less than 30.75) and normosmia (equal or above 30.75) [26].

For *retronasal testing* the Candy-Smell test (CST) was administered [12, 13]. The CST has been validated with 23 candies, containing 500 mg sorbitol and the one targeted aroma, and applied in clinical routine in a forced-choice manner with visual and verbal cues. After placing the candy on the tongue, subjects were asked to suck or chew the candy and rinse their mouth with water after each candy. The maximal attainable test score was 23 (each identified candy yielding 1 point).

### Flavor education and training

Slides (Microsoft^®^ PowerPoint^®^), mobile computer devices and sagittal anatomy illustrations of the human nose and mouth (showing odor routes) were used to demonstrate key points of flavor perception. All involved sensory modalities, including retronasal olfactory and trigeminal chemosensory perception, as well as visual, acoustic and tactile stimuli (e.g., appearance, texture) in food “capturing” were discussed, leaving enough time for questions. This session lasted for approximately 45 min.

FT subjects were provided with a “flavor protocol” including a list of 50 flavors: common ingredients, various spices fruits and herbs were chosen according to presumable easy availability. During training period, the subjects were instructed to consciously experience all flavors (“with all senses”) from the list on separate days, especially those they were interested in. Given a training period of 16 weeks, time was provided to try one flavor from the list of 50 flavors every second day and fill out a short questionnaire on each experience (see Table 2). Flavors had to be rated on how much the ingredient met subject’s imagination or memory (1 = no at all, 10 = completely meets my imagination/memory). This was used to monitor participation compliance. Patients were encouraged to repeat “tasting” of various flavors to ensure daily training sessions. Since flavor exposure can hardly be prohibited, group A was allowed to continue FT (re-enjoy suggested flavors) if desired.

**Table 2:** Flavor protocol (example)

Flavor	Perception through	
*Goat cheese*	Seeing—color	*Yes*
	Seeing—appearance	*Yes*
Date: *01/18/2012*	Seeing—SHAPE	*Yes*
Time: *07:03 p.m*	Hearing	*No*
	Temperature	*No*
	Texture	*Yes*
	Smell	*No*
Overall meets my i/m	Not 1-2-3-*X*-5-6-7-8-9-10 completely	

To increase participation compliance and consciousness to perception of selected flavors, subjects were asked to keep records (i.e., fill out this protocol on each flavor). An overall score was obtained on how much the flavor of the ingredient met subject`s imagination or memory (i/m). Italic letters: exemplary completed protocol

### Self-assessment

Subjects had to rate their abilities to smell (subjective assessment of smell, SAS) and perceive detailed flavors during eating and drinking (subjective assessment of flavor, SAF) like wine and herbs on a ten-point scale (1 = no smell/flavor, 10 = excellent smell/flavor perception) before psychophysical testing.

### Dietary changes and quality of life

To detect changes in food preference, we applied the established dietary alterations score (DAS) at V1, V3 and V5. As initially proposed, 26 questions on changes in food and beverage intake with an answer-pattern “more”/“less”/“no changes” were summed to a comparable score of a maximum of 26 points: “no change” receives 0 points, “more” and “less” 1 point [2]. As a health-related quality-of-life (QoL) measure the SF-36 was applied [30, 31]. All these measurements were obtained three times throughout the study period (at V1, V3 and V5).

### Statistical analysis

GraphPrism 8.1.2 (GraphPad Software, La Jolla, CA, USA) was used for statistical analysis and data visualization. One-way repeated measures ANOVA (rm-ANOVA) were conducted to compare the effect of session on orthonasal (TDI) as well as retronasal olfactory performance (CST) in all patients in session 1 (V1), session 2 (V3) and session 3 (V5) followed by Tukey's post hoc test. One-way repeated measures ANOVA (rm- ANOVA) were also conducted to compare the effect of group on orthonasal (TDI; and its subtests: T, D and I) as well as retronasal olfactory performance (CST) in flavor training starting after 1 week (group A) and after 18 weeks (delayed start; group B) condition followed by Tukey's post hoc test. Wilcoxon matched-pair tests as well as Mann–Whitney tests were performed to assess group differences. The normality of data was tested using Shapiro–Wilk test. Correlational analyses were performed using the Pearson correlation coefficient (*r*). The *p* value was set at < 0.05.

## Results

### Findings at study enrolment

Retronasal olfactory function (CST) correlated significantly with orthonasal function (TDI) in overall subjects (*r*_29_ = 0.50; *p* = 0.005), with scores showing best correlation with the Sniffin Sticks subtest I (*r*_29_ = 0.46; *p* = 0.012). Two anosmic subjects scored slightly (15 and 16) above the proposed CST cut-off for anosmia of 13 [12, 13]. With regard to subjective ratings at V1: SAS and SAF did not correlate significantly with chemosensory test results, except for SAF and the CST in group B showing an inverse correlation (*r*_14_ = − 0.79, *p* = 0.001). For results per group at V1 see Table 1.

### Flavor education and training

All subjects attended the FE session and reported useful, as well as novel information was presented. Overall, only one subject (Group B) dropped out of trial after visit 3 (after 17 weeks) due to personal reasons. FT compliance was high: in average participants tried 30.4 ± 12.9 (median: 33) flavors from the list of 50 suggestions and returned protocols on their experience (for an example see Table 2). Two subjects did not try any suggested flavors and one subject only tried one flavor. However, they stated to consciously having tried flavors other than from the list during the training period.

**Fig. 2:**
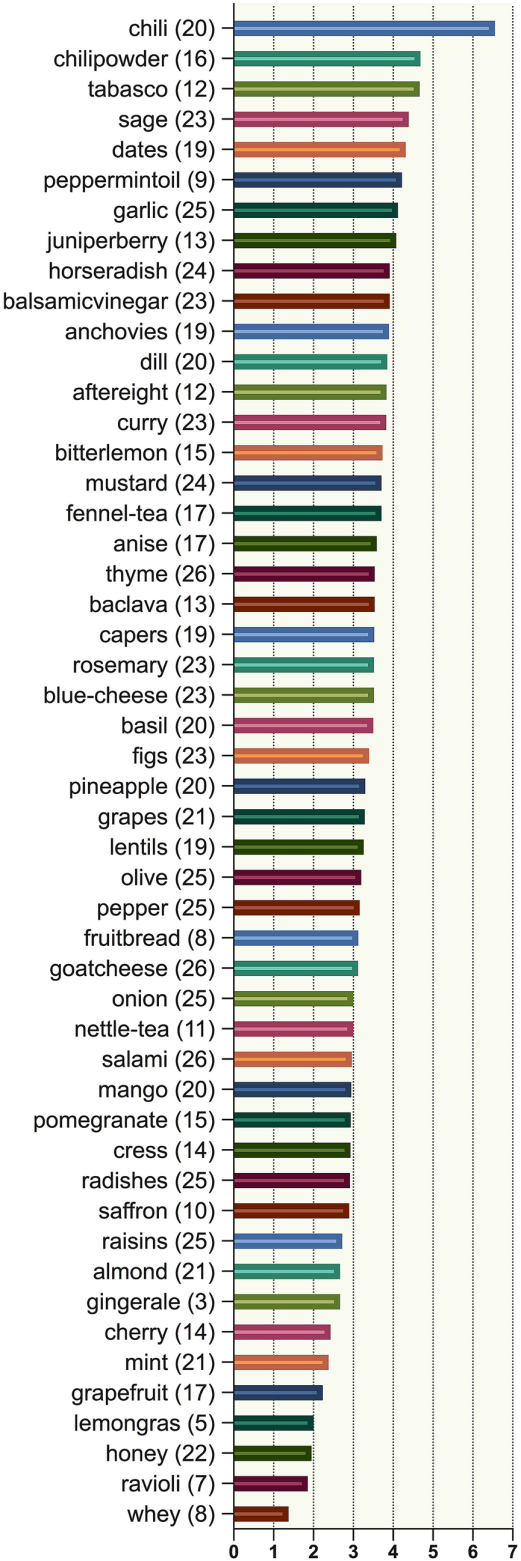
Illustration to which extend flavors met the imagination and memories of participants in average on a ten-point scale with higher scores reflecting flavors being more as anticipated. Notable, flavors with stronger gustatory and/or trigeminal components (i.e., spicy, peppermint, very sweet) scored higher. Numbers in brackets represent how many participants tried suggested flavor. For instance, ginger-ale was only tried by 3, while goatcheese was tried by 26 participants

Figure 2 illustrates to which extent flavors met the imaginations on flavors. On average, the participants stated to focus on visual (29.0 ± 3.8 cases of enjoyed flavors), odor (31.7 ± 5.4), temperature (34.2 ± 5.0) and texture (34.8 ± 5.4) aspects. Acoustic aspects were only important in 12.7 ± 2.6 flavors/dishes.

### Overall test results over time

Overall TDI scores from V1 to V5 improved in 10 (34.5%) patients (6 of group A, 4 of group B) in a clinically significant way, as proposed by Gudziol et al. for the TDI score (> 5.5) [32]. For all patients, the mean improvement in TDI score was 4.0 ± 3.3. TDI difference (V5–V1) correlated weakly significant with changes in CST scores (*r*_28_ = 0.40; *p* = 0.035). Two (6.9%) patients scored slightly worse on TDI at V5 in comparison to V1 (− 1.0 and − 3.25, respectively).

Overall TDI differed significantly: rm-ANOVA showed a significant effect of session (timepoint) on TDI [*F* (2, 28) = 15.90, *p* < 0.0001]. Tukey post hoc comparisons revealed a significant difference between V1(22.7 ± 6.4)/V5(27.0 ± 5.8), *p* < 0.0001 and V3(23.9 ± 7.1)/V5(27.0 ± 5.8), *p* = 0.0025. The differences of TDI scores obtained during V3 and V1 (V3–V1) of group A and B were not significantly different (*p* = 0.294, Mann–Whitney test).

For group A rm-ANOVA showed a significant effect of session (timepoint) on TDI [*F* (2, 14) = 8.824, *p* = 0.0037]. Tukey post hoc comparisons revealed a significant difference between V1(23.3 ± 6.7)/V5(27.6 ± 6.2), *p* = 0.0009 and V3(23.7 ± 7.1)/V5(27.6 ± 6.2), *p* = 0.0033. For group B rm-ANOVA showed a significant effect of session (timepoint) on TDI [*F* (2, 13) = 11.59, *p* = 0.0003]. Tukey post hoc comparisons revealed a significant difference between V1(22.6 ± 6.0)/V3(25.2 ± 6.0), *p* = 0.0064 and V1(22.6 ± 6.0)/V5(26.3 ± 5.5), *p* = 0.0021.

### Retronasal test results over time

**Fig. 3:**
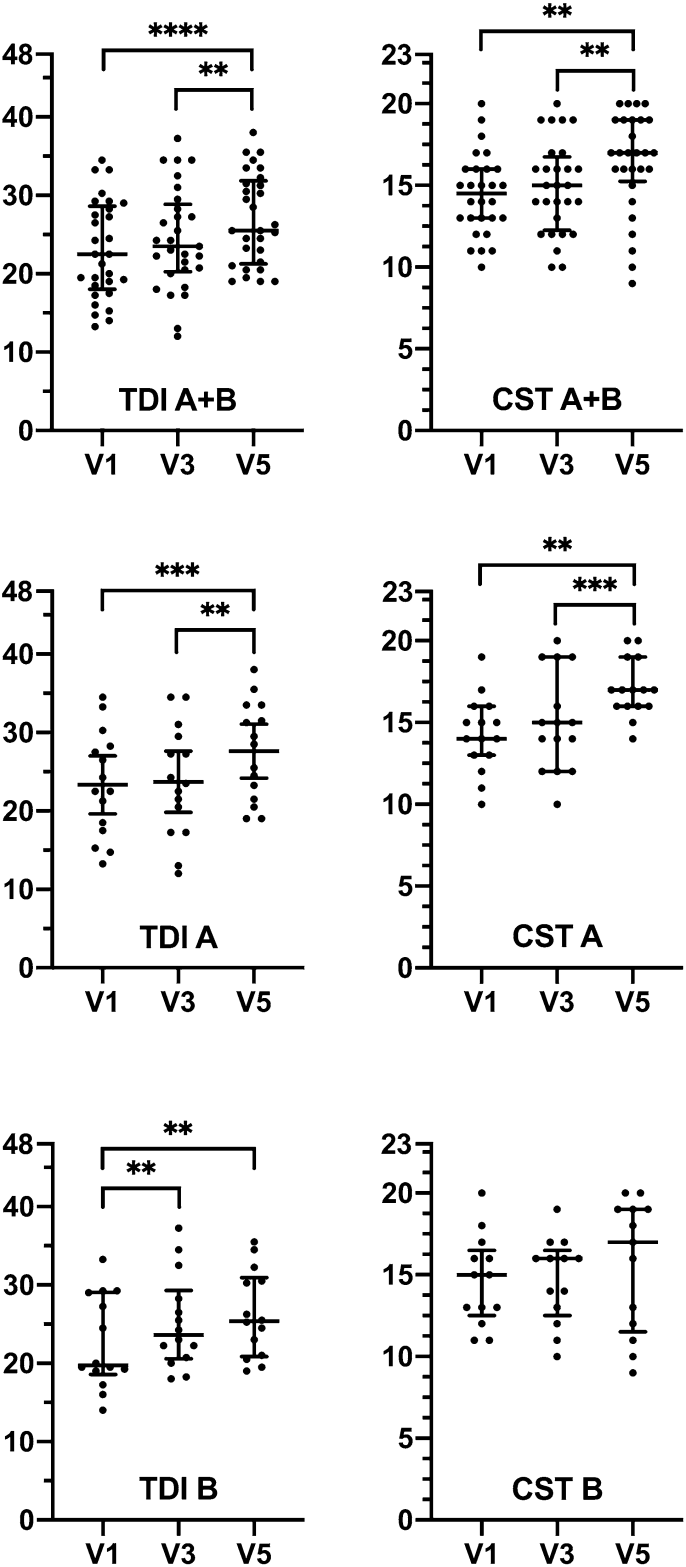
Scatter-dot plots of orthonasal (TDI) and retronasal tests (CST) per groups (A/B). Lines show medians (Q0.5) and interquartile ranges (Q.25, Q.75); Outliers are shown as individual data points. V1: 1st TDI testing at enrolment and after 17 weeks (visit 3 = V3) and after 34 weeks (V5). (**) *p* < .01; (***) *p* < .001; (****) *p* < .0001

Overall CST results differed significantly: rm-ANOVA showed a significant effect of session (timepoint) on CST [*F* (2, 27) = 8.063, *p* = 0.0018]. Tukey post hoc comparisons revealed a significant difference between V1(14.3 ± 2.6)/V5(16.4 ± 3.1), *p* = 0.0065 and V3(14.8 ± 2.8)/V5(16.4 ± 3.1), *p* = 0.0017. For group A rm-ANOVA showed a significant effect of session (timepoint) on CST [*F* (2, 14) = 9.012, *p* = 0.0052]. Tukey post hoc comparisons revealed a significant difference between V1(14.3 ± 2.3)/V5(17.1 ± 1.8), *p* = 0.0021 and V3(15.1 ± 3.1)/V5(17.1 ± 1.8), *p* = 0.0007). For group B rm-ANOVA showed no significant effect of session (timepoint) on CST [*F* (2, 12) = 1.082, *p* = 0.3386]. Figure 3 visualizes data on TDI and CST results, also per group with significant results as indicated.

### Age, duration of impairment and self-assessment

No associations were found between age/duration of OD (DurOD) and TDI/CST differences (V5-V1) (Age/TDI: *r*_29_ = − 0.1758; Age/CST: *r*_29_ = − 0.008812; DurOD/TDI: *r*_29_ = − 0.007059; DurOD/CST: *r*_29_ = − 0.1045; all *p* > 0.05). SAS differed significantly for group A from V1 (3.1 ± 1.9) to V5 (4.3 ± 2.4) (*p* = 0.0334, Wilcoxon test) and group B from V1 (2.8 ± 1.0) to V5(4.8 ± 1.7) (*p* = 0.0005, Wilcoxon test). SAF group A did not differ significantly (*p* > 0.05), group B however did from V1 (5.5 ± 2.3) to V5 (6.7 ± 1.6) (*p* = 0.0156, Wilcoxon test).

### Mental/physical health and dietary changes

Obtained health-related QoL measurements (SF-36) at V1, V3 and V5 did not differ significantly from each other (*p* > 0.05), indicating stable health conditions of participants throughout the trial period.

The mean overall dietary alteration score was 6.0 ± 6.5 (range 0–19), being even lower than Aschenbrenner et al. found in normosmic subjects [2]. There was no significant difference for the overall DAS of V1, V3 and V5 (*p* > 0.05, Wilcoxon test) and between groups (A vs. B) at V1, V3 and V5 (*p* > 0.05, Mann–Whitney test). Assessing test–retest reliability, DAS scores of V1 and V3 correlated significantly (*r*_30_ = 0.96; *p* < 0.0001), as well as of V1 and V5 (*r*_28_ = 0.93; *p* < 0.0001).

## Discussion

The present investigation provided the following major results: (1) FE was greatly appreciated by participating patients as reflected by direct responses and the very low drop-out rate in this fairly time-consuming trial. (2) FT was accepted broadly among this cohort: most of the suggested flavors were tried and protocols revealed interesting findings of ratings on perception, showing that flavors with high trigeminal components more likely met the expectations (see Fig. 2). (30 Chemosensory abilities improved subjectively and semi-objectively (as measured by TDI and CST) following FT. In one-third of enrolled subjects the TDI score improved by > 5.5 (which also was considered as a significant improvement in previous studies on OT [33]) and orthonasal olfactory improvement was in general accompanied by retronasal olfactory improvement as measured by the CST. These changes were not affected by age and duration of OD.

With regard to latter findings (changes in chemosensoric abilities) there are limitations that need to be addressed. During the first 17 weeks (with one group performing and one group not performing FT) no group differences in improvements of the CST were found and TDI scores improved in group B. Over 34 weeks, however, only group A (performing FT longer) showed improvements in retronasal abilities as measured by CST and improvements in TDI scores were more prominent than in group B. Despite this, group differences over the full 34 weeks can only be interpreted with caution due to the lack of a control group and we just see this as a positive trend. Noteworthy, this was not the primary aim of the trial. This study was intended to implement FE and most importantly investigate for possible strategies on how FT can be offered to patients to ensure participation compliance.

As another limitation, sample size was not large (as within the nature of a pilot study); however, only slightly lower than in the initial study on OT [18]. Studies on perceptual learning in the chemical senses appear to be notoriously underpowered [34] and larger sample sizes are needed, as well as application of a “OT alone versus OT and FT”-study design to evaluate effects of FT.

All subjects received systemic glucocorticoids as initial treatment (before enrolment) and performed an unmonitored olfactory training without subjective improvement. This is a frequent inconvenient situation in smell and taste clinics and second-line therapies to offer are awaited by clinicians. Recent findings suggest OT to be driven “top-down”, hence coming from central processes [35]. Furthermore, same odors were found to activate different central structures when applied retronasally versus orthonasally and retronasal odor perception stimulates many brain regions including gustatory circuits [36, 37]. It hence seems appealing to also use the retronasal route for conscious stimulation and structured training.

In this pilot study on FT we chose to offer a list of 50 flavors to respect a diverse diet and not dictate to eat, e.g., mustard or chili every day. Also, we wanted to raise curiosity of patients by adding also rather unusual (but available) ingredients to the list. Possibly this approach supported shown compliance. Future investigators may also cut down the list to the most frequently used ingredients and studies are certainly needed to standardize training protocols. Another issue which will have to be considered: the CST, as well as other published retronasal olfactory tests, still lacks a definition on clinical meaningful changes (which has been established for the TDI [32]). In this study, shown CST improvements were not accompanied by improvements in subjective ratings of flavor. This underlines also a need for studies on subjective and measured flavor perceptive abilities.

Subjective flavor ratings were surprisingly high in this cohort and possibly in consequence no apparent changes in dietary behavior due to OD were found in this cohort. Noteworthy, the DAS showed high test–retest reliability, which supports the usage of this questionnaire in other fields of research on dietary changes.

Since all included patients were of German mother tongue, it feels necessary to point out an important linguistic circumstance: various languages, including German, do not supply a commonly used distinction between “taste” and “flavor” [38]. This seems to potentiate misunderstandings in everyday clinical routine. OD patients used to report poor management of their disease, as Landis et al. published in 2009 [39]. A decade later, going hand in hand with increasing awareness for benefits of OT, clinicians seeking a proper work-up and therapy of OD patients, will certainly have to mention the principals of flavor perception. Limited time resources in a clinical setting however restrict a comprehensive educational “lesson”. Applied simple presentation strategies can be of great value in a quick, but ample outpatient care.

Whereas patients mainly report OD correctly, unnoticed anosmia is a frequent finding [40]. In the present cohort, subjective ratings did not correspond to orthonasal test results and 3 patients sought medical help due to olfactory complaints, but then were tested within normative ranges. Nonetheless, FT was appreciated and completed by two of these subjects. Self-reported OD with normosmic test results is an occasional clinical finding with a decreasing probability with age [41]. It also can be postulated that prior to complaint onset, these subjects had ranged at higher percentiles of normative datasets and dropped down to still normosmic ranges (which is why we did not exclude these subjects for analysis).

Sensory education has been advocated in children with increasing willingness to eat healthier and to try novel foods [42, 43]. In elderly reduced olfactory function can lead to a monotonous diet [44]. At the same time OT can be applied in elderly and improve quality of life [45]. Therefore, the scope of FE and FT is not limited to OD alone and further investigations can be valuable in diverse settings/diseases.

## Conclusion

We demonstrate flavor education is feasible and appreciated in a clinical setting. Conscious retronasal stimulation in terms of a flavor training seems to be a welcomed second-line therapy in patients with olfactory dysfunction. This study shows beneficial trends of FT, however further studies with larger sample sizes and standardized training protocols are needed.
